# Rush or relax: migration tactics of a nocturnal insectivore in response to ecological barriers

**DOI:** 10.1038/s41598-022-09106-y

**Published:** 2022-03-23

**Authors:** Michiel Lathouwers, Tom Artois, Nicolas Dendoncker, Natalie Beenaerts, Greg Conway, Ian Henderson, Céline Kowalczyk, Batmunkh Davaasuren, Soddelgerekh Bayrgur, Mike Shewring, Tony Cross, Eddy Ulenaers, Felix Liechti, Ruben Evens

**Affiliations:** 1 Research Group: Zoology, Biodiversity and Toxicology, Centre for Environmental Sciences, Hasselt University, Campus Diepenbeek, Agoralaan, Gebouw D, 3590 Diepenbeek, Belgium; 2 Department of Geography, Institute of Life, Earth and Environment (ILEE), University of Namur, Rue de Bruxelles 61, 5000 Namur, Belgium; 3 British Trust for Ornithology, The Nunnery, Thetford, Norfolk, IP24 2PU UK; 4 Wildlife Science and Conservation Centre, Ulaanbaatar, Mongolia; 5 Department of Biology, Mongolian National University of Education, Ulaanbaatar, Mongolia; 6 School of Biosciences, Cardiff University, Cardiff, UK; 7 MPS Ecology, Heol y Cyw, Bridgend, UK; 8 Llandrindod Wells, Wales, UK; 9 Agentschap Natuur en Bos, Regio Noord-Limburg, Herman Teirlinck Havenlaan 88 bus 75, 1000 Brussels, Belgium; 10 Swiss Ornithological Institute, Seerose 1, 6204 Sempach, Switzerland; 11 Max Planck Institute for Ornithology, Eberhard-Gwinner-Straße, 82319 Seewiesen, Germany; 12 Department of Biology, Behavioural Ecology and Ecophysiology, University of Antwerp, Universiteitsplein 1, 2610 Wilrijk, Belgium

**Keywords:** Animal migration, Behavioural ecology

## Abstract

During their annual migration, avian migrants alternate stopover periods, for refuelling, with migratory flight bouts. We hypothesise that European Nightjars (*Caprimulgus europaeus*) adapt their daily migration tactics in association with biomes. We tracked the autumn migration of 24 European Nightjars, from breeding populations in Mongolia, Belgium and UK, using GPS-loggers and multi-sensor data loggers. We quantified crepuscular and nocturnal migration and foraging probabilities, as well as daily travel speed and flight altitude during active migration in response to biomes. Nightjars adopt a rush tactic, reflected in high daily travel speed, flight altitude and high migration probabilities at dusk and at night, when travelling through ecological barriers. Migration is slower in semi-open, hospitable biomes. This is reflected in high foraging probabilities at dusk, lower daily travel speed and lower migration probabilities at dusk. Our study shows how nightjars switch migration tactics during autumn migration, and suggest nightjars alternate between feeding and short migratory flight bouts within the same night when travelling through suitable habitats. How this may affect individuals’ fuel stores and whether different biomes provide refuelling opportunities *en route* remains to be investigated, to understand how future land-use change may affect migration patterns and survival probabilities.

## Introduction

Many animals show seasonal cyclical movement patterns between key habitats located thousands of kilometres apart. This behaviour allows migratory species to exploit high quality and abundant resources during different periods of the annual cycle, maximizing their survival and/or reproductive success^1^. Because long-distance migrations are energetically demanding^2–4^, it is critical that individuals balance their available energy reserves *en route*. Hence, migrants will attempt to optimize their energy budgets in response to resource availability by employing a range of different migration tactics^5^.

Among long-distance migratory birds, many species alternate stopover periods, for refuelling, with migratory flight bouts^6^. An individual’s migration schedule is most likely endogenously controlled, but fine-tuned by external cues^7^. During migratory flight bouts, flight tactics can be shaped by resource availability and refuelling opportunities^8–10^, which in turn is determined by environmental drivers, such as local weather conditions^11^, topography^12^ and habitat^13^. When refuelling opportunities are limited, for example in deserts, over extended water crossings or ice sheets^14^, individuals may choose to undertake longer flight bouts and travel faster with unidirectional movements to minimize the time spent in these unsuitable areas^15^. Conversely, migrants may minimize energy costs by undertaking shorter, slower movements when suitable habitats for refuelling are available^15^. Additionally, avian migrants further minimize their energy expenditure by optimizing their flight altitude^16^, in response to wind, temperature, topography and other factors^17^. This optimization involves a trade-off between energy spent on forward movement against climbing to a specific altitude at the beginning of a flight bout In order to reduce the total cost of transport by selecting an altitude with favourable conditions^18^. The majority of empirical studies on individuals’ flexibility in migratory flight behaviour is focused on large-bodied migratory species, able to carry heavy tracking devices. Several species of raptors, for example, have been shown to migrate rapidly over unsuitable ecological barriers and travel slower in more suitable areas^10,13,19–21^.

The miniaturization of biologging technologies have revolutionized animal tracking, allowing research on endogenous factors (e.g., behaviour, physiology, and orientation) and external factors (e.g., ambient temperature, barometric pressure) influencing bird migration for even the smallest migratory passerines and near-passerine species^22,23^. Here, we aim at investigating how biomes affect migratory flight tactics in a small-bodied (< 100 g), crepuscular insectivore, the European Nightjar (*Caprimulgus europaeus*, hereafter referred to as nightjar). We hypothesise that individuals spend less time per day on active migration (i.e. relaxed migration) in biomes which may provide foraging opportunities at dusk or dawn. Conversely, we expected more time to be invested in active migration (i.e., rushed migration) in biomes where such foraging opportunities are lacking. Using multi-sensor loggers of five individual nightjars, we quantified daily migration and foraging probabilities during dusk, night, and dawn as well as daily altitude change. Additionally, using archival GPS-logger data we quantified daily travel speed and flight altitude. Investigating how these migration parameters are associated with biomes will provide us with insights into individual migration tactics in response to the landscape.

## Methods

### Field methods

We conducted fieldwork in Mongolia (48.6° N, 110.8° E; 2018), Belgium (51.1° N, 5.5° E; 2015–2019) and the UK (52.5° N, 0.7° E; 2015–2018, 53.1° N, − 3.5° E; 2018–2019). These sites were selected to include individuals from longitudinal extremes of the global distribution range of the species. We captured nightjars in breeding areas using ultra-fine mist nets (Ecotone, 12 × 3 m) and tape lures^24^, marked each individual with a unique alphanumeric ring and fitted a data logger dorsally between the wings^24,25^. In total, we tagged 114 adult individuals, with 1.8 g Pathtrack Ltd. nanoFix or Biotrack Ltd. PinPoint-40 archival GPS-logger (Belgium and the UK) or a 1.2 g SOI-GDL3pam^26^ multi-sensor logger (Mongolia and Belgium; hereafter multi-sensor logger; for a list of deployments see Supplementary Materials Table S1). The multi-sensor loggers contain sensors that recorded air pressure, ambient light intensity, air temperature and acceleration in 5-min intervals and magnetic field in 4-h intervals. The GPS-loggers record longitude, latitude, and altitude at 24-h intervals at 00:00 GMT, when the birds were likely to be in flight (for specific settings see Table S1).

### Estimation of migration routes from multi-sensor loggers

From the recovered multi-sensor loggers, we estimated migration routes by combining flight activity data with light measurements. Multi-sensor loggers measure flight activity as the sum of the absolute differences in acceleration along the z-axis^27^, which allows the detection of flapping flights^26^. As such, we used an automated k-means classification algorithm to classify activity data into three categories: inactivity, low and high activity. This allowed us to define migratory flight bouts as periods (minimum 60 min) of uninterrupted high activity and the delineation of stopovers as periods of one day or longer where migration was interrupted, i.e. where no migratory flight activity was recorded^28^.

We analysed light measurements using a threshold method to provide daily position estimates for the migration period (*sensu*^29^) with the R-package PAMLr^28^. Initial position estimates were obtained by identifying sunset and sunrise events from the light-intensity data. We used Hill-Ekstrom calibration, based on light-intensity measurements from the sedentary wintering period, to model the error in defining these sunrise/sunset events caused by shading of the light sensor^30^. Initial position estimates during periods of stopover were grouped together and treated as a single location. Final position estimates were obtained using an Estelle model in SGAT^31^ to refine initial location estimates based on Markov chain Monte Carlo (MCMC) simulations and provide a probability distribution around each estimate (two locations per day). In this model we included the following priors: the location where the tags were deployed, the model describing the error in twilight times, a distribution of probable flight speeds (relaxed gamma distribution of shape = 2.2 and rate = 0.08) and a spatial probability mask where stationary positions over water were not possible. We ran this model for 250 iterations to initiate, before tuning the model with the aforementioned priors (three runs with 300 iterations). Finally, to obtain the final position estimates the model was run for 2000 iterations to ensure convergence. Subsequently, we calculated the 97.5% credible intervals around these final position estimates.

### Calculation of migration parameters

We only considered migratory flight bouts during autumn migration because most loggers only partially recorded spring migration. Autumn migration was defined as the period between the departure at the breeding areas and the arrival at the wintering areas^25^. From the multi-sensor loggers, we excluded data from stopover days. For days containing migratory flight bouts, activity data (inactivity, low activity, high activity, migrating) were categorized into two binary variables: “migrating” (yes/no) and “activity” (yes/no). From previous studies, we know that high and low activity levels are likely associated with foraging behaviour^25^. Hence, “activity” will subsequently be referred to as “foraging activity”. For each position estimate, information on the timing of day, night, and twilight (i.e., sunset and sunrise) were extracted using the “classify_DayTime” function from the R-package RchivalTag^32^. We subdivided the daily activity into four time groups: day (from sunrise to sunset), night (from astronomical dusk to astronomical dawn) dusk (from sunset to astronomical dusk) and dawn (from astronomical dawn to sunrise).

Air pressure data recorded by the multi-sensor loggers, measured in hectopascals, were converted to estimated flight altitudes above sea level using the atitudeCALC function in the PAMLr R-package^28^, according to the International Standard Atmosphere model (International Organization for Standardization 1975: ISO 2533:1975) utilising the following formula:

$$ H = - \frac{{T_{0} }}{L} \times \left( {1 - \left( {\frac{P}{{P_{0} }}} \right)^{{\frac{1}{5.2561}}} } \right) $$

where H = flight altitude above sea level (m), T_0_ = temperature at sea level (K), L = lapse rate (temperature change per meter increase in altitude, deg/m), P = pressure recorded by the sensor (HPa), P_0_ = pressure at sea level (HPa). We used the standard assumptions of P_0_ = 1013.25 HPa, T_0_ = 288.15 K and L = – 0.0065 deg/m^33^. These data on estimated flight altitude above sea level were subsequently used to calculate daily altitude change by subtracting the daily maximum and minimum estimated flight altitude above sea level.

GPS tracks were visually inspected and periods wherein directional movement was interrupted for at least one day were removed, in order to retain only travel segments^34^. From GPS data, we calculated daily travel speed, defined as the distance travelled between consecutive observed locations in 24 h interval periods (km/day). Second, we calculated flight altitude above ground level by subtracting the elevation of the landscape from the recorded altitude. The altitude of the landscape was derived from the CleanTOPO 30 arc second global bathymetric and terrestrial elevation dataset^35^.

### Grouping data according to biome

To analyse differences in migration tactics in response to biomes, a global classification of natural communities in a particular region based on dominant or major vegetation types and climate^36,37^, we extracted information from the Terrestrial Ecosystems of the World dataset version 2.0 (Dataset published by World Wildlife Fund) for each position estimate and GPS observation. GPS observations were assigned to biomes using the point sampling tool in QGIS version 3.10.10^38^. To account for the spatial inaccuracy of geolocator position estimates^29^, we determined the most abundant biome in the 97.5% credible interval around each position estimate using the zonal statistics tool in QGIS.

The width of the 97.5% credible interval around geolocation position estimates on active migration days was on average 21.23 ± 9.01 degrees in latitude and 9.30 ± 2.14 degrees in longitude. While these credible intervals are large, the most abundant biome represented on average 61.73 ± 15.34% of the surface area. In the East Atlantic flyway, the following biomes were the most abundant in the credible intervals around the different position estimates: “Temperate Broadleaf and Mixed Forests”, “Mediterranean Forest, Woodland and Scrub”, “Desert and Xeric Shrubland”, “(Sub)Tropical Grassland, Savannah and Shrub” and “(Sub)Tropical Moist Broadleaf Forest”. In the East Asia/East African flyway these were: “Temperate Grassland, Savannah and Shrub”, “Montane Grassland and Savannah”, “Desert and Xeric Shrubland” and “(Sub)Tropical Grassland, Savannah and Shrub”. Two position estimates from the East Asia/East African Flyway, one categorized as “Boreal Forest” and one as “(Sub)Tropical Moist Broadleaf Forest” were excluded from further analyses since these were the only recorded position estimates in these biomes. In further analyses we distinguished between two categories of biomes, namely ecological barriers, and hospitable biomes (*sensu*^39^). The ecological barriers include the “Desert and Xeric scrub” and the “Mediterranean Forest, Woodland and Scrub” biome as well as the “Tropical Moist Broadleaf Forest” biome which is presumed to be a soft ecological barrier for migrating nightjars^34^. Other biomes consist of semi-open habitats and are presumed hospitable biomes.

### Statistical analyses

From daily activity data, we assessed the probability that individuals were migrating in a particular period of the night in response to biome categories. We constructed a binomial model with migratory activity (binary; migrating or not migrating) as the outcome variable and biome category (binary; hospitable or barrier) and period (categorical: dusk, night and dawn) as the predictor. We also included activity of the previous time step to control for temporal autocorrelation. Subsequently we constructed the same model with foraging activity (binary; foraging or not foraging) as outcome variable. Additionally, we investigated how biome category influenced daily altitude change by constructing a similar model with daily altitude change as outcome variable.

From the GPS data, we assessed variation in daily travel speed and flight altitude in response to biome category. We constructed models containing either daily travel speed or flight altitude as the outcome variable and biome category as the predictor. The flight altitude model included a zero-inflation parameter to allow modelling the probability of excess zeros in the conditional part of the model. To investigate the influence of specific biomes on migratory activity, foraging activity, daily altitude change, daily travel speed and flight altitude, we constructed a second set of models where the predictor of biome category was replaced with the variable containing information on the individual biome (Supplementary Materials).

Individual identity was included in all models as a random intercept. Models were fitted using Generalized Linear Mixed Models (GLMMs) with maximum likelihood using the R package glmmTMB version 1.0.2.1^40^. Model misspecification problems were checked using the package DHARMa version 0.3.3.0.^41^ and when required a zero-inflated model was fitted to model the probability of excess zeros in the conditional part of the model. The significance of the models was established using a likelihood ratio test (Anova with a ‘Chisq’ test statistic). We then used post-hoc Tukey’s comparisons to test the significance of the difference between pairs of interactions between the main predictors using the R package emmeans version 1.5.1.^42^.

## Ethics approval and consent to participate

The Mongolian and Belgian research protocols were approved by the Mongolian (Ministry of Environment and Tourism, license numbers: 06/2564 and 06/2862) and Belgian (Agency for Nature and Forest, license numbers: ANB/BL-FF/V18-00086 and ANB/ BL-FF/19-00087-VB) authorities. In the UK, use of GPS tags was approved by the Special Methods Technical Panel and licensed by the British Trust for Ornithology. All protocols were carried out in accordance with the relevant guidelines and regulations.

## Results

We recovered 30 loggers (two multi-sensor loggers in Mongolia, four multi-sensor and nine GPS-loggers in Belgium, and 15 GPS-loggers in the UK; all from males; Supplementary Materials S1) constituting a recovery rate of 26.3%. While comparable to other studies (23%;^34^) this low recovery rate is probably caused by the late deployment of loggers on non-resident individuals with no fidelity to the study site in Belgium (late August 2018) and a low recovery success due to bad weather conditions during a two-week trapping session in Mongolia (July 2019). Of the 30 recovered loggers, six (one multi-sensor logger from Belgium and five GPS-loggers from the UK) did not contain usable data due to a technical failure.

**Figure 1:**
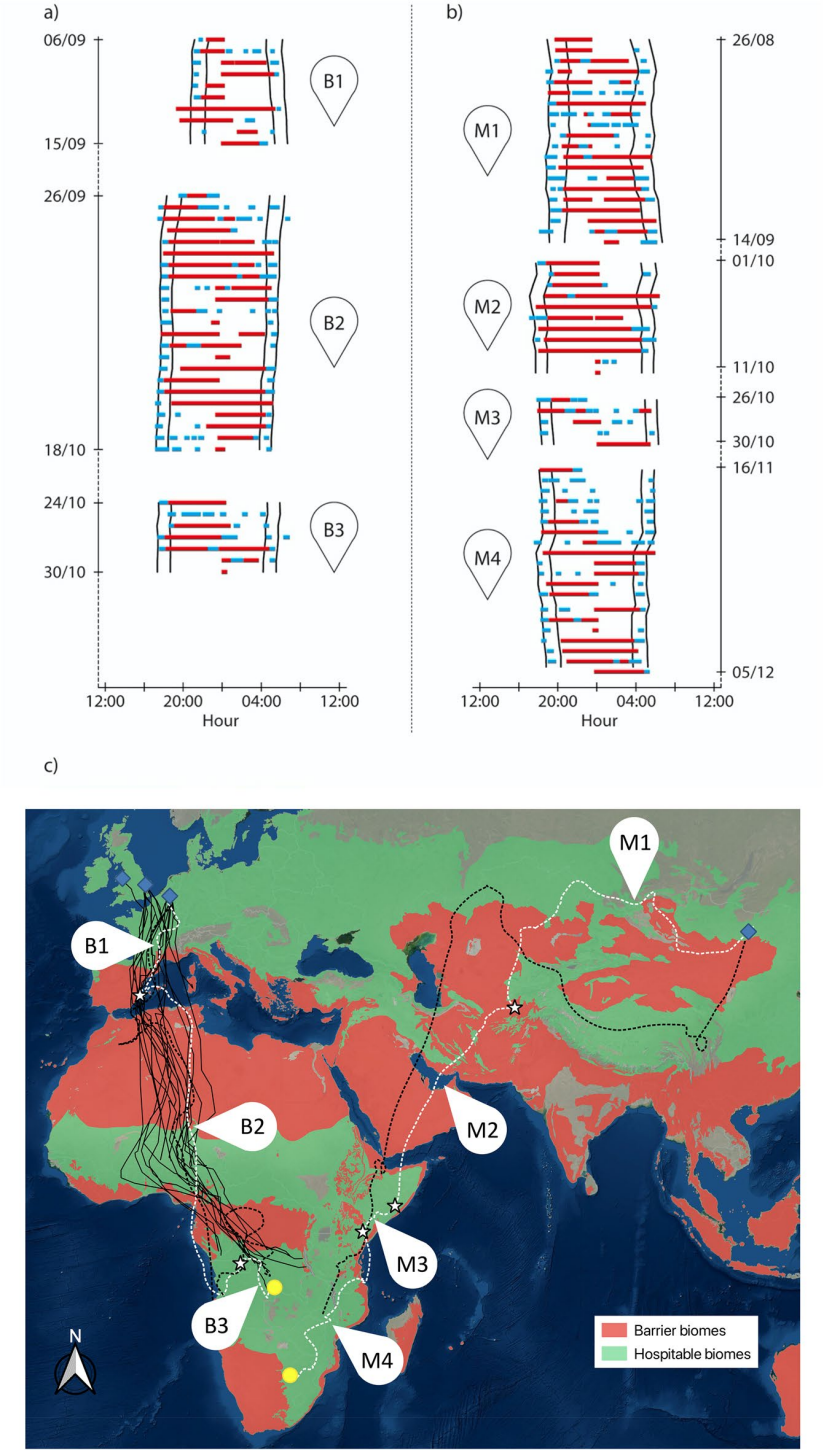
Actograms (**a**, **b**) and migration routes (**c**) of European Nightjars breeding in Belgium (**a**) and in Mongolia (**b**). Example of actograms from two individuals showing daily activity (red = migratory flight, blue = foraging activity, white = no/low activity, measured per five-min period) covering migration segments (Belgian individual: B1-3, Mongolian individual: M1-4) from dusk until dawn. Each horizontal bar shows one day with time on the X-axis. Time is plotted in eight-hour intervals and centred around local midnight. Black lines show the timing of sunset at the estimated location of each individual. Stopover periods are not shown on the actograms. The bottom map (**c**) shows the categorized biomes [barriers (red), hospitable (green); adapted from^37^] with geolocator tracks (dotted lines) and GPS-tracks (full lines). White dotted lines show the migration routes of one Belgian individual (represented in actogram **a**) and one Mongolian individual (represented in actogram **b**). White stars indicate stopover areas (> 3 days), blue diamonds represent breeding areas and yellow circles represent wintering areas.

Tracked nightjars from Western Europe travel to their wintering grounds in Central Africa along the East Atlantic flyway, with stopovers in Southern Europe, Northern Africa, and the Sahel region (Fig. 1c). Nightjars breeding in Eastern Mongolia migrate to wintering areas in South-Africa and Zimbabwe along the East Asia/East African flyway, with stopovers in Central Asia, the horn of Africa and East Africa (Fig. 1c). The two Mongolian nightjars travelled a minimum migration distance (distance between stopovers) of on average 11696 ± 487 km and spent an average of 100 ± 1.4 days between departure at the breeding site and arrival at the wintering site. Nightjars migrating from Western Europe spent 62 ± 8.1 days on autumn migration, travelling a minimum migration distance of 8633 ± 887 km.

**Figure 2:**
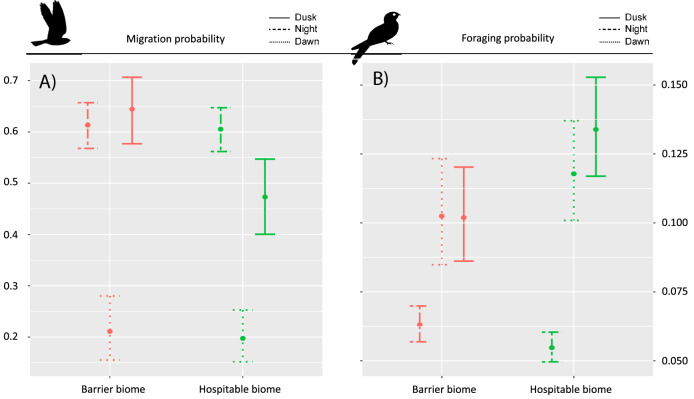
Differences in migration (**A**) and foraging probability (**B**) in barrier (red) or hospitable (green) biomes during dusk (solid line), night (dashed line) and dawn (dotted line). n = 53,556 5-min intervals; 5 individuals. Shown are model estimates and their 95% confidence intervals based on the model results in Table 1.

Flight activity data show that nightjars’ migration tactics differ between biomes they are traveling through and the period during which they are traveling. There is strong evidence that migration probability during dusk is significantly higher during the crossing of ecological barriers when compared to the category of biomes presumed to be hospitable (Fig. 2, Table 1). Migration probability during the night and dawn periods is not different between biome categories (Fig. 2, Table 1). Our data additionally show evidence that the probability of foraging activity during dusk is higher in hospitable biomes compared to foraging probabilities in ecological barriers (Fig. 2, Table 1). Conversely, nocturnal and dawn foraging probabilities did not differ between biome categories (Fig. 2, Table 1). (for full model results see Supplementary Materials Table S3).

**Table 1:** Results of post-hoc Tukey’s comparisons between barrier and hospitable biome categories based on binomial GLMMs where the effect of biome category was tested on migration probability and foraging probability during dusk, night and dawn (n = 53,556 5-min intervals; 5 individuals).

	Dusk					Night					Dawn				
Outcome variable	Estimate	SE	df	t.ratio	p.value	Estimate	SE	df	t.ratio	p.value	Estimate	SE	df	t.ratio	p.value
Migration probability	0.702	0.174	25,051	4.026	0.001	0.034	0.071	25,051	0.471	0.997	0.083	0.224	25,051	0.373	0.999
Foraging probability	− 0.309	0.112	25,051	− 2.750	0.066	0.150	0.060	25,051	2.500	0.124	− 0.157	0.129	25,051	− 1.213	0.831

Positive estimates refer to a positive effect of the barrier biome category.

**Figure 3:**
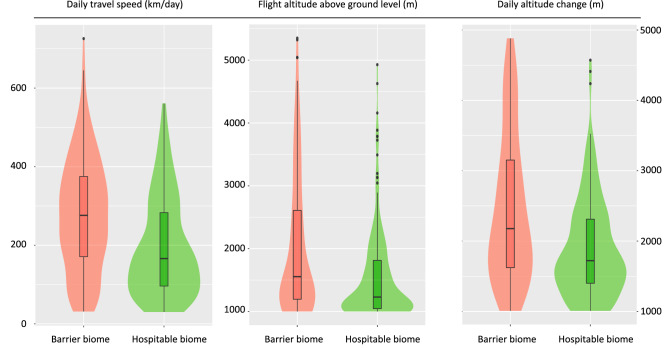
Box plots and violin plots showing (**A**) daily travel speed (n = 408 days; 19 individuals), (**B**) flight altitude above ground level (n = 408 days; 19 individuals), (**C**) daily altitude change (n = 338 days; 5 individuals) in relation to biome category. Violin plot: probability density. Box plot: median (thick black line), 25% and 75% quantiles (thin box), 90% range (whiskers) and outliers.

GPS data and barometric pressure recordings show how ecological barriers are crossed at significantly higher speed and altitude. Our GPS data show strong evidence that nightjars cover larger distances per day (Barrier mean = 275 km/day, Hospitable mean = 195 km/day) and fly at higher altitude above the ground (Barrier mean = 1168 m AGL, Hospitable mean = 576 m AGL) when traversing ecological barriers (Fig. 3, Table 2). Similarly, barometric pressure data show strong evidence that daily altitude change is higher during the crossing of ecological barriers (Barrier mean = 1426 m, Hospitable mean = 864 m) compared to hospitable biomes (Fig. 3, Table 2). (For full model results see Supplementary Materials Table S4).

**Table 2:** Results of post-hoc Tukey’s comparisons between barrier and hospitable biome categories based on binomial GLMMs where the effect of biome category was tested on (a) travel speed and flight altitude based on GPS observation (n = 408; 19 individuals) and (b) daily altitude change based on multi-sensor logger data (n = 338 days; 5 individuals).

Outcome variable	Estimate	SE	df	t ratio	*p* value
**(a) GPS data**					
Daily travel speed	80.2	13.3	404	6.033	< 0.001
Flight altitude	592	98.3	366	6.020	< 0.001
**(b) Multi-sensor data**					
Daily altitude change	562.065	91.405	334	6.149	< 0.001

Positive estimates refer to a positive effect of the barrier biome category.

## Discussion

During autumn migration, nightjars rush through ecological barriers while migration intensity is relaxed in more hospitable biomes containing semi-open habitats. Rush migration reflects high daily travel speed, high flight altitude and high migration probabilities at dusk. Relaxed migration is characterised by high foraging probability at dusk, lower daily travel speed, lower flight altitude and lower migration probabilities at dusk, suggesting that nightjars may forage and perform short migratory flight bouts within the same night.

A more detailed analysis of migration tactics within specific biomes (Supplementary Materials) shows that Nightjars in our study rush through the Sahara and the Arabian deserts, which are considered ecological barriers for avian migrants in the Palearctic—African migration system^43^. This rushed migration tactic seems to indicate that nightjars attempt to minimize the time spent in these unfavourable areas (sensu optimal migration theory;^6^). This confirms the findings of a previous study based on a subset of the GPS tracks represented here, where unequal migration speeds where found throughout the flyway^34^. High daily travel speed is also observed in the Central African Rainforest (CAR), supporting the idea that, although interspersed with open areas of human settlements and farmland, the dense tropical rainforest may also form an ecological barrier for Eurasian migratory birds^34,44,45^.

Our data further suggest that nightjars migrate slower in biomes containing semi-open habitats, such as savannahs, grasslands and shrublands. Nightjars’ daily travel speed is lower and are interspersed with periods of inactivity and possible foraging behaviour (Fig. 1a,b). It suggests that nightjars may opportunistically feed during the crepuscular hours when they encounter hospitable biomes, which may provide insectivorous birds with relatively large amounts of food^46^. This suggests that nightjars, when encountering semi-open habitats, may travel short distances between sequential sites and accumulate small amounts of extra fuel, a few hours per day. This may correspond to ample observations of nightjars feeding rapidly in key foraging habitats at dusk and dawn during the breeding period^25,47^. At this point it is unclear whether the combination of foraging and migrating reflects a “hop” tactic (i.e. short distance migrations to predictable and reliable food resources^48^) or a “fly-and-forage” tactic (i.e. combine foraging with moving in the direction of travel^19,21,49^). Nevertheless, both tactics have been observed in combination with a more conventional stopover tactic (i.e. “jump” migration *sensu*^50,51^) for other larger-bodied Palaearctic avian migrants. Osprey (*Pandion haliaetus*)^13,21^ and Eleonora’s Falcon (*Falco eleonorae*)^19^, for example, relax their pace across potential foraging habitat in order to combine daily migrating with refuelling, while stopping over for prolonged periods before and after crossing ecological barriers. The acquired fuel reserves en route can therefore serve as an insurance to reduce the time spent at stopover sites before or after crossing ecological barriers^52,53^.

To limit inferences from our data with limited spatial accuracy^30^, which is inherent to the applied geolocation method^54^, we accounted for the error margins around position estimates in assigning environmental characteristics to position estimates and we opted to investigate large scale biomes as opposed to environmental data with a finer spatial resolution. Nevertheless, we acknowledge this method is not without potential biases in evaluating the relationship between environmental characteristics and migration tactics. Future studies investigating how environmental factors influence migration ecology of aerial migrants would therefore benefit from employing a multi-scale approach^55^ which combines tracking technologies with fine spatial resolutions such as GPS-tracking^56^.

In accordance with our findings based on data from multi-sensor loggers, GPS-observations in our study indicate nightjars adjust their daily travel speed and flight altitude in response to biomes. GPS-tracked individuals migrating from Western Europe cover approximately 320 km per day across ecological barriers while more suitable areas are crossed at approximately 200 km per day, reflecting how the interplay of topography, wind patterns and food availability influence travel speed of nightjars^57,58^. The observed travel speeds are in line with previous studies on nightjars^34,58,59^ and are similar to those of other insectivorous long-distance migrants such as Common Cuckoo (*Cuculus canorus*) and Common Swift (*Apus apus*)^60^. Migratory birds are known to adapt not only travel speed but also flight altitude in order to reduce energetic costs and water loss during migration^61^. During the autumn crossing of the Sahara, lower altitudes offer favourable tailwinds^62^ and many migratory species, including nightjars, may therefore fly at low altitudes in order to minimize energy costs instead of minimizing water loss^63^. We observed a mean flight altitude of approximately 1.7 km above the ground when individuals crossed the Sahara or Arabian Desert, similar to other recent studies investigating flight altitude of small nocturnal migrants using barometer logging^26,33,64^. This indicates that when nightjars cross ecological barriers with long flight bouts they climb to higher altitudes, providing better convective cooling, a lack of turbulence and reduced air density^65^, which allows them to achieve higher flight speeds. The lower flight altitude we observe in hospitable biomes may be related to the shorter duration of flight bouts, meaning nightjars were more likely to be at or near ground level during the night when GPS fixes were taken, but also confirm our findings that migrating nightjars are less likely to spend time on active migration when traversing hospitable biomes.

## Conclusions

Nightjars adopt variable migrations tactics during active autumn migration. Ecological barriers, where opportunities for foraging are limited, are crossed at a higher pace. This allows individuals to avoid risks and minimize the time they spend in these inhospitable areas. In contrast, where food resources are available, nightjar seemingly spend less time migrating per day while foraging efforts are increased. The ability to opportunistically exploit available resources in between active migratory flight, in our view, could serve to (partially) negate unforeseen poor foraging opportunities en route. While this study provides a first look into how nightjars balance active migration with potential refuelling outside of stationary stopover periods, in future research we will investigate how these different migration tactics during active migration relate to behaviour during prolonged stopovers, allowing investigations on the impact of global land use change on individual migration patterns and tactics.

## Supplementary Information

Supplementary Information.

## Data Availability

The datasets used in this study are available from the corresponding author on reasonable request.

## Abbreviations

GPS: Global positioning system
HPa: Hectopascal
GIS: Geographic information system
UK: United Kingdom
CAR: Central African Rainforest
GLMM: Generalized linear mixed model
AGL: Above ground level
ASL: Above sea level
