# Supplementary material for: Rush or relax: migration tactics of a nocturnal insectivore in response to ecological barriers
Source: Sci Rep. 2022 Mar 23;12:4964. doi: 10.1038/s41598-022-09106-y (PMC8943004; doi:10.1038/s41598-022-09106-y)
Supplement: Supplementary file 1 — Supplementary Information. [file 41598_2022_9106_MOESM1_ESM.docx]

**Supplementary Materials: Rush or relax: migration tactics of a nocturnal insectivore in response to ecological barriers.**

Michiel Lathouwers ^1,2*^, Tom Artois ^1^, Nicolas Dendoncker ^2^, Natalie Beenaerts ^1^, Greg Conway ^3^, Ian Henderson ^3^, Céline Kowalczyk ^1^, Batmunkh Davaasuren ^4^, Soddelgerekh Bayrgur ^5^, Mike Shewring ^6,7^, Tony Cross ^8^, Eddy Ulenaers ^9^, Felix Liechti ^10^ and Ruben Evens ^11,12^

^1^ Hasselt University, Centre for Environmental Sciences, Research Group: Zoology, Biodiversity and Toxicology, Campus Diepenbeek, Agoralaan, Gebouw D, 3590 Diepenbeek, Belgium.

^2^ University of Namur, Department of Geography, Institute of Life, Earth and Environment (ILEE), Rue de Bruxelles 61, 5000 Namur, Belgium.

^3^ British Trust for Ornithology, The Nunnery, Thetford, Norfolk IP24 2PU, UK.

^4^ Wildlife Science and Conservation Centre, Ulaanbaatar, Mongolia.

^5^ Department of Biology, Mongolian National University of Education, Ulaanbaatar, Mongolia.

^6^ Cardiff University, School of Biosciences, Cardiff, UK.

^7^ MPS Ecology, Heol y Cyw, Bridgend, UK.

^8^ Consultant Ornithologist, Llandrindod Wells, Wales, UK.

^9^ Agentschap Natuur en Bos, Regio Noord-Limburg, Herman Teirlinck Havenlaan 88 bus 75, 1000 Brussels, Belgium.

^10^ Swiss Ornithological Institute, Seerose 1, 6204 Sempach, Switzerland.

^11^ Max Planck Institute for Ornithology, Eberhard-Gwinner-Straße, 82319 Seewiesen, Germany.

^12^ University of Antwerp, Department of Biology, Behavioural Ecology and Ecophysiology, Universiteitsplein 1, 2610 Wilrijk, Belgium

* Corresponding author: [michiel.lathouwers@uhasselt.be](mailto:michiel.lathouwers@uhasselt.be), 0032/472.358.303

**Influence of specific biomes on migration parameters**

A more detailed analysis of daily flight speed, based on GPS observations, shows that nightjars travel faster in “Desert and Xeric Shrubland” (mean= 347.12 km/day) and “(Sub)Tropical Moist Broadleaf Forest” (mean= 277.08 km/day) in comparison to “(Sub)Tropical Grassland, Savannah and Shrubland” (mean= 176.11 km/day), “Temperate Broadleaf and Mixed Forests” (mean= 224.80 km/day) and “Mediterranean Forest, Woodland and Scrub” (mean= 206.79 km/day) (Table S2). In the same way, average flight altitude above ground level was higher in “Desert and Xeric Shrubland” (mean= 1747.5 m AGL) when compared to Mediterranean Forest, Woodland and Scrub” (mean= 517.8 m AGL) , “Temperate Broadleaf and Mixed Forest” (mean= 327.1 m AGL) and “(Sub)Tropical Grassland, Savannah and Scrub” (mean= 641.4 m AGL) (Table S2).

**Supplementary tables**

Table S1 Summary of tracking device deployment and capture/recapture data.

| **Country** | **Longitude** | **Latitude** | **Year** | **Device** | **Deployments** | **Recoveries** | **Containing usable data** | **Interval** |
| --- | --- | --- | --- | --- | --- | --- | --- | --- |
| Belgium | 5.5°E | 51.1°N | 2016-2019 | GPS | 21 | 9 | 9 | 24h |
| Belgium | 5.5°E | 51.1°N | 2018-2019 | SOI-GDL3pam | 25 | 4 | 3 | / |
| Mongolia | 110.8°E | 48.6°N | 2018 | SOI-GDL3pam | 20 | 2 | 2 | / |
| UK | 0.7°E | 52.5°N | 2015-2017 | GPS | 48 | 15 | 10 | 24h/48h |

Table S2 Results of post-hoc Tukey’s comparisons between biomes based on binomial GLMMs where the effect of biome was tested on daily travel speed and flight altitude of 19 European Nightjars from Belgium and UK during autumn migration

|  | **Flight Altitude** | | | | | **Daily Travel Speed** | | | | |
| --- | --- | --- | --- | --- | --- | --- | --- | --- | --- | --- |
|  | **estimate** | **SE** | **df** | **t.ratio** | **p.value** | **estimate** | **SE** | **df** | **t.ratio** | **p.value** |
| Temperate Broadleaf and Mixed Forest - Temperate Conifer Forest | -734.935 | 479.452 | 360 | -1.533 | 0.789 | -151.836 | 73.531 | 398 | -2.065 | 0.440 |
| Temperate Broadleaf and Mixed Forest - Mediterranean Forest, Woodland and Scrub | -291.525 | 144.177 | 360 | -2.022 | 0.468 | 18.012 | 20.325 | 398 | 0.886 | 0.987 |
| Temperate Broadleaf and Mixed Forest - Desert and Xeric Shrubland | -1492.929 | 146.647 | 360 | -10.180 | **<0.001** | -122.320 | 20.894 | 398 | -5.854 | **<0.001** |
| Temperate Broadleaf and Mixed Forest - (Sub)Tropical Grassland, Savannah and Shrubland | -333.196 | 131.127 | 360 | -2.541 | 0.182 | 48.693 | 17.879 | 398 | 2.723 | 0.119 |
| Temperate Broadleaf and Mixed Forest - Flooded Grassland and Savannah | -755.897 | 479.172 | 360 | -1.578 | 0.764 | -102.578 | 73.531 | 398 | -1.395 | 0.859 |
| Temperate Broadleaf and Mixed Forest - Mangrove | -465.059 | 581.743 | 360 | -0.799 | 0.993 | -109.010 | 89.470 | 398 | -1.218 | 0.926 |
| Temperate Broadleaf and Mixed Forest - (Sub)Tropical Moist Broadleaf Forest | -482.011 | 181.211 | 360 | -2.660 | 0.139 | -52.283 | 25.139 | 398 | -2.080 | 0.430 |
| Temperate Conifer Forest - Mediterranean Forest, Woodland and Scrub | 443.409 | 480.649 | 360 | 0.923 | 0.984 | 169.848 | 73.475 | 398 | 2.312 | 0.290 |
| Temperate Conifer Forest - Desert and Xeric Shrubland | -757.994 | 481.276 | 360 | -1.575 | 0.765 | 29.516 | 73.634 | 398 | 0.401 | 1.000 |
| Temperate Conifer Forest - (Sub)Tropical Grassland, Savannah and Shrubland | 401.739 | 476.074 | 360 | 0.844 | 0.990 | 200.529 | 72.836 | 398 | 2.753 | 0.110 |
| Temperate Conifer Forest - Flooded Grassland and Savannah | -20.962 | 660.419 | 360 | -0.032 | 1.000 | 49.259 | 101.942 | 398 | 0.483 | 1.000 |
| Temperate Conifer Forest - Mangrove | 269.876 | 741.433 | 360 | 0.364 | 1.000 | 42.826 | 113.975 | 398 | 0.376 | 1.000 |
| Temperate Conifer Forest - (Sub)Tropical Moist Broadleaf Forest | 252.924 | 493.048 | 360 | 0.513 | 1.000 | 99.553 | 74.949 | 398 | 1.328 | 0.888 |
| Mediterranean Forest, Woodland and Scrub - Desert and Xeric Shrubland | -1201.403 | 148.766 | 360 | -8.076 | **<0.001** | -140.332 | 20.697 | 398 | -6.780 | **<0.001** |
| Mediterranean Forest, Woodland and Scrub - (Sub)Tropical Grassland, Savannah and Shrubland | -41.671 | 133.771 | 360 | -0.312 | 1.000 | 30.682 | 17.648 | 398 | 1.739 | 0.662 |
| Mediterranean Forest, Woodland and Scrub - Flooded Grassland and Savannah | -464.371 | 479.736 | 360 | -0.968 | 0.979 | -120.589 | 73.475 | 398 | -1.641 | 0.725 |
| Mediterranean Forest, Woodland and Scrub - Mangrove | -173.533 | 582.382 | 360 | -0.298 | 1.000 | -127.022 | 89.424 | 398 | -1.420 | 0.848 |
| Mediterranean Forest, Woodland and Scrub - (Sub)Tropical Moist Broadleaf Forest | -190.485 | 182.812 | 360 | -1.042 | 0.968 | -70.295 | 24.975 | 398 | -2.815 | 0.094 |
| Desert and Xeric Shrubland - (Sub)Tropical Grassland, Savannah and Shrubland | 1159.732 | 129.762 | 360 | 8.937 | **<0.001** | 171.014 | 18.301 | 398 | 9.344 | **<0.001** |
| Desert and Xeric Shrubland - Flooded Grassland and Savannah | 737.032 | 481.300 | 360 | 1.531 | 0.790 | 19.743 | 73.634 | 398 | 0.268 | 1.000 |
| Desert and Xeric Shrubland - Mangrove | 1027.870 | 581.393 | 360 | 1.768 | 0.642 | 13.310 | 89.555 | 398 | 0.149 | 1.000 |
| Desert and Xeric Shrubland - (Sub)Tropical Moist Broadleaf Forest | 1010.918 | 180.939 | 360 | 5.587 | **<0.001** | 70.037 | 25.441 | 398 | 2.753 | 0.110 |
| (Sub)Tropical Grassland, Savannah and Shrubland - Flooded Grassland and Savannah | -422.700 | 477.244 | 360 | -0.886 | 0.987 | -151.271 | 72.836 | 398 | -2.077 | 0.432 |
| (Sub)Tropical Grassland, Savannah and Shrubland - Mangrove | -131.863 | 577.883 | 360 | -0.228 | 1.000 | -157.704 | 88.900 | 398 | -1.774 | 0.638 |
| (Sub)Tropical Grassland, Savannah and Shrubland - (Sub)Tropical Moist Broadleaf Forest | -148.814 | 165.918 | 360 | -0.897 | 0.986 | -100.976 | 23.029 | 398 | -4.385 | **<0.001** |
| Flooded Grassland and Savannah - Mangrove | 290.838 | 741.548 | 360 | 0.392 | 1.000 | -6.433 | 113.975 | 398 | -0.056 | 1.000 |
| Flooded Grassland and Savannah - (Sub)Tropical Moist Broadleaf Forest | 273.886 | 493.913 | 360 | 0.555 | 0.999 | 50.295 | 74.949 | 398 | 0.671 | 0.998 |
| Mangrove - (Sub)Tropical Moist Broadleaf Forest | -16.952 | 591.498 | 360 | -0.029 | 1.000 | 56.727 | 90.639 | 398 | 0.626 | 0.999 |

Table S3 Results of generalized mixed-effect models showing effects of biome category on nocturnal and crepuscular migration- and foraging activity as well as daily altitude difference of 5 European nightjars from Belgium and Mongolia during autumn migration

| **Migration activity** | | | | |
| --- | --- | --- | --- | --- |
| **Predictors** | **estimate** | **SE** | **Z value** | **P value** |
| Intercept | -4.50230 | 0.19659 | -22.90 | <2e-16 |
| Biome Category  (Hospitable) | -0.08350 | 0.22366 | -0.37 | 0.7089 |
| Period  (Dusk) | 1.91335 | 0.21408 | 8.94 | <2e-16 |
| Period  (Night) | 1.78007 | 0.18295 | 9.73 | <2e-16 |
| Previous activity | 6.13658 | 0.06522 | 94.09 | <2e-16 |
| Biome Category  (Hospitable) : Period (Dusk) | -0.61867 | 0.28320 | -2.18 | 0.0289 |
| Biome Category  (Hospitable) : Period (Night) | 0.04982 | 0.23458 | 0.21 | 0.8318 |
| **Random effect** | **Variance** | **SD** |  |  |
| Individual Identity | 0.03109 | 0.1763 |  |  |
| **Foraging activity** | | | | |
| **Predictors** | **estimate** | **SE** | **Z value** | **P value** |
| Intercept | -2.489754 | 0.107311 | -23.20 | <2e-16 |
| Biome Category  (Hospitable) | 0.156507 | 0.129062 | 1.21 | 0.2253 |
| Period  (Dusk) | -0.006024 | 0.133557 | -0.05 | 0.9640 |
| Period  (Night) | -0.528625 | 0.109824 | -4.81 | 1.48e-06 |
| Previous activity | 3.024005 | 0.050401 | 60.00 | < 2e-16 |
| Biome Category  (Hospitable) : Period (Dusk) | 0.152349 | 0.170902 | 0.89 | 0.3727 |
| Biome Category  (Hospitable) : Period (Night) | -0.306268 | 0.142099 | -2.16 | 0.0311 |
| **Random effect** | **Variance** | **SD** |  |  |
| Individual Identity | 0.005046 | 0.07104 |  |  |
| **Daily altitude difference** | | | | |
| **Predictors** | **estimate** | **SE** | **Z value** | **P value** |
| Intercept | 1425.74 | 118.83 | 11.998 | < 2e-16 |
| Biome Category | -562.07 | 91.41 | -6.149 | 7.79e-10 |
| **Random effect** | **Variance** | **SD** |  |  |
| Individual Identity | 46320 | 215.2 |  |  |

Table S4 Results of generalized mixed-effect models showing effects biome category on daily travel speed and flight altitude of 19 European Nightjars from Belgium and UK during autumn migration

| **Daily Travel Speed** | | | | |
| --- | --- | --- | --- | --- |
| **Predictors** | **estimate** | **SE** | **Z value** | **P value** |
| Intercept | 275.409 | 9.761 | 28.215 | < 2e-16 |
| Biome Category | -80.191 | 13.293 | -6.033 | 1.61e-09 |
| **Random effect** | **Variance** | **SD** |  |  |
| Individual Identity | 4.809e-04 | 0.02193 |  |  |
| **Flight Altitude** | | | | |
| **Predictors** | **estimate** | **SE** | **Z value** | **P value** |
| Intercept | 1168.22 | 97.41 | 11.99 | <2e-16 |
| Biome Category | -592.07 | 98.35 | -6.02 | 1.74e-09 |
| **Zero inflation model** | | | | |
| Intercept | -2.2647 | 0.1781 | -12.71 | <2e-16 |
| **Random effect** | **Variance** | **SD** |  |  |
| Individual Identity | 71391 | 267.2 |  |  |
